# Hardware Demonstration of SRDP Neuromorphic Computing with Online Unsupervised Learning Based on Memristor Synapses

**DOI:** 10.3390/mi13030433

**Published:** 2022-03-11

**Authors:** Ruiyi Li, Peng Huang, Yulin Feng, Zheng Zhou, Yizhou Zhang, Xiangxiang Ding, Lifeng Liu, Jinfeng Kang

**Affiliations:** School of Integrated Circuits, Peking University, Beijing 100871, China; 1901111210@pku.edu.cn (R.L.); fengyulin@pku.edu.cn (Y.F.); zhouzime@pku.edu.cn (Z.Z.); 1801111298@pku.edu.cn (Y.Z.); xxding@pku.edu.cn (X.D.); kangjf@pku.edu.cn (J.K.)

**Keywords:** spike-rate-dependent plasticity (SRDP), online unsupervised learning, neuromorphic computing, memristor

## Abstract

Neuromorphic computing has shown great advantages towards cognitive tasks with high speed and remarkable energy efficiency. Memristor is considered as one of the most promising candidates for the electronic synapse of the neuromorphic computing system due to its scalability, power efficiency and capability to simulate biological behaviors. Several memristor-based hardware demonstrations have been explored to achieve the capacity of unsupervised learning with the spike-rate-dependent plasticity (SRDP) learning rule. However, the learning capacity is limited and few of the memristor-based hardware demonstrations have explored the online unsupervised learning at the network level with an SRDP algorithm. Here, we construct a memristor-based hardware system and demonstrate the online unsupervised learning of SRDP networks. The neuromorphic system consists of multiple memristor arrays as the synapse and the discrete CMOS circuit unit as the neuron. Unsupervised learning and online weight update of 10 MNIST handwritten digits are realized by the constructed SRDP networks, and the recognition accuracy is above 90% with 20% device variation. This work paves the way towards the realization of large-scale and efficient networks for more complex tasks.

## 1. Introduction

The human brain is a highly efficient system, which consists of approximately 10^11^ neurons and 10^15^ synapses with merely 20 W power consumption [1,2,3]. Neuromorphic computing is a new computing paradigm inspired by the brain, with the advantage of massive parallelism and distributed storage, and is claimed as a promising technology to enhance information analysis abilities in the data-rich era [4,5,6,7]. However, existing hardware demonstrations are far from competing with the biological ones in terms of efficiency and power consumption [8,9,10]. One reason is that the systems are constructed based on CMOS devices with complex synapses and neuron circuits occupying quite a large area [11,12,13]. Therefore, the compact nanoelectronic device which can successfully simulate the biological elements is essential to construct efficient networks [2]. Recently, the memristor with high density, low power consumption and tunable conductance has shown great promise for the synapses [14,15,16,17]. Another attribution to the inefficiency is that the recognition tasks are realized via supervised learning, which demands a large amount of training data and additional feedback circuits, leading to time latency and energy consumption [18,19,20,21], especially when online training is required [22,23,24]. Thus, recent studies focus on unsupervised learning [25,26,27,28,29,30], where the synaptic weights are usually updated according to bio-inspired local learning rules [31,32,33], such as spike-timing-dependent plasticity (STDP) [34,35] and spike-rate-dependent plasticity (SRDP) [36]. STDP refers to the learning principle that relative timing between pre-synaptic and post-synaptic spikes determines the direction of weight update and the magnitude of weight change [37,38,39]. SRDP is another learning rule that modulates the synaptic weights by the frequency of pre- and post-neuron activities, which is one of the most critical learning algorithms for neuromorphic computing [40,41,42].

Early research studies have proved that memristor devices can exhibit SRDP-like behaviors, including SiO_x_N_y_:Ag-based diffusive memristor [43], HfO_x_-based memristor [44], TiO_x_/AlO_y_-based [45] oxide memristor, AgInSbTe-based chalcogenide memristor [46], hybrid CMOS/memristor structure [26,28], and devices with many other materials [47,48,49]. Going beyond the device demonstrations, several hardware implementations of pattern learning by SRDP have been proposed [26,28]. Milo et al. demonstrated online unsupervised learning of patterns with 8 × 8 pixels by SRDP based on the 4T1R structure [26]. Nevertheless, the learning ability of the small-scale network is limited, which is unable to accomplish challenging tasks, such as classification of different inputs and recognition of data sets. Recently, Huang et al. proposed a single-layer fully-connected network to classify 10 images by SRDP and constructed a CNN-SRDP network to recognize the whole MNIST images with up to 92% accuracy, which enlarges the learning abilities [4]. However, only simulation results are presented, and the device demonstration is performed based on discrete cells. Therefore, hardware demonstration by SRDP at the network level is of great importance to address more practical tasks [2,50,51,52].

In this work, we present a neuromorphic hardware system, which is comprised of multiple memristor arrays, DACs, ADCs and many other assemblies, and is equipped with inference and training functions. The SRDP characteristic is implemented experimentally by the memristor synapses and CMOS neurons. A 196 × 10 SRDP neural network is constructed to demonstrate the online unsupervised learning of 10 MNIST digits, and about 90% classification accuracy is achieved.

## 2. Memristor-Based Neuromorphic Hardware System

**Figure 1:**
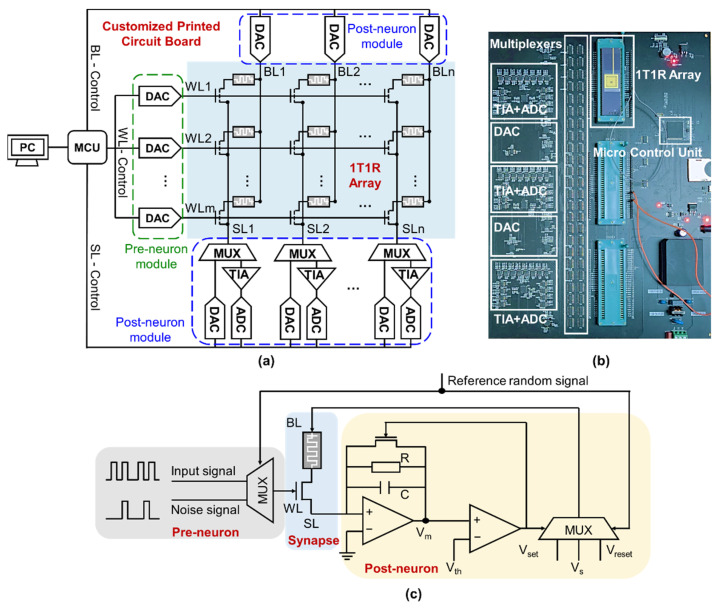
Memristor-based neuromorphic system. (**a**) Circuit diagram of the system; (**b**) Photograph of the customized printed circuit board; (**c**) Circuit diagram of the memristor synapse and the corresponding CMOS neurons.

Here, a memristor-based neuromorphic system is constructed for the hardware demonstration of SRDP neural networks. The system consists of three parts, including memristor crossbar arrays, the customized printed circuit board (PCB) and the personal computer (PC), as shown in Figure 1a. The memristor array provides hardware synapses, and the device conductance is considered as the analogy of synaptic weight. The vector-matrix multiplications and weight update can be performed on the array. The PCB implements partial functions of the neurons, which primarily consists of Digital-Analog Converters (DACs), trans-impedance amplifier (TIA), Analog-Digital Converters (ADCs) and multiplexers (MUX), as shown in Figure 1b. DACs in the pre-neuron module are used to generate input signal and noise signal to WL of the array under the control of a reference random signal. The post-neuron is made up of an integrator, a comparator and a multiplexer, as can be seen in Figure 1c. Therefore, DACs in the post-neuron module are used to generate constant voltage for inference tasks and spike pulses to BL or SL for weight update. ADCs together with TIAs are used to read the integral current across the synapses through SL. MUXs are utilized to select different memristor chips and operation modes including inference and weight update. MCU controls the discrete components and processes data. Matlab script running on PC is used to control the generation of signals and perform some calculations of the leaky-integrate-and-fire (LIF) post-neuron, including the accumulation of membrane voltage (V_m_) and the comparison between V_m_ and threshold voltage (V_th_). The computer sends control commands and communicates with MCU via a serial port.

**Figure 2:**
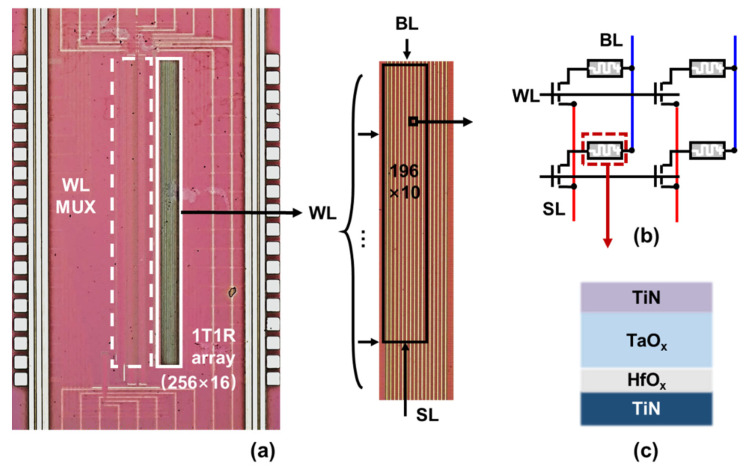
Memristor chip. (**a**) The micrograph of the 256 × 16 memristor array; (**b**) The structure of the crossbar array; (**c**) Schematic illustration of the memristor cell.

Figure 2a shows the micrograph of the memristor chip. Each packaged chip is integrated with 256 × 16 1T1R cells and multiplexers to control and select word lines. The crossbar array is constructed by connecting the gates of transistors in the same row (WL) and the top electrodes (TE) of memristors in the same column (BL). The sources of the transistor are wired to the same SL, which is parallel to BL, as can be seen in Figure 2b. The structure of the array is designed to meet the requirements for the SRDP algorithm, where input signals of the pre-neurons are sent to WL and the top electrodes of the memristor corresponding to the same post-neuron should be connected for synchronous weight update. Figure 2c shows the memristor device with TiN/TaO_x_/HfO_x_/TiN structure. TiN is used as the bottom electrode, on the top of which an 8-nm HfO_2_ resistive layer was deposited by atomic layer deposition (ALD) at 250 °C. Then, a 45-nm TaO_x_ was deposited as a capping layer by magnetron sputtering with an Ar/N_2_ atmosphere. The TiN top electrode is grown by physical vapor deposition and patterned by the dry etching method.

## 3. Memristor Synapse with SRDP Characteristic

**Figure 3:**
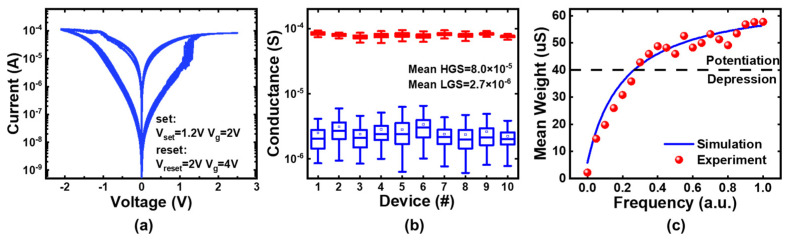
Basic properties and SRDP characteristics of the memristor. (**a**) The I-V characteristics; (**b**) The conductance distribution of ten devices; (**c**) Measured and simulated SRDP characteristic.

The basic properties and SRDP characteristics of the memristor are shown in Figure 3. The typical I-V characteristic is presented in Figure 3a. The distribution of high conductance state (HGS) and low conductance state (LGS) of ten memristors selected randomly is shown in Figure 3b. The result shows that HGS is around 80.0 μS and LGS is 2.7 μS on average, indicating approximately 30× conductance window. The variation of HGS is below 20% and that of LGS is about 80%. The SRDP learning rules and the circuit of memristor synapse and CMOS neurons have been illustrated in Section 2. To prove the feasibility of the SRDP algorithm, we perform experiments based on the hardware system. According to the previous work, the learning efficiency and accuracy are sensitive to the circuit parameters of the post-neuron [4]. Thus, we should select the circuit parameters firstly, which include the leaky resistance R, the capacitor C of the integrator, the threshold voltage V_th_ of the comparator in the post-neuron module, and so on. The various signals are initiated as the binarized sequences with certain probabilities, where a high level “1” represents a spike with 1 μs width and “0” represents that there is no spike generated. We randomly select a device in the array for the demonstration of SRDP behavior. Initially, the device has the probability P_g_ = 0.5 to be in HGS. The training process of SRDP is comprised of three stages, including accumulation, potentiation and depression. When training starts, the system first enters the accumulation stage. DAC in the pre-neuron module generates V_g_ according to the input signal and sends it to the selected WL, while that in the post-neuron module sends small constant voltage (V_s_) to BL. When the transistor of the memristor synapse is switched on, the current will be generated following Ohm’s law and read out by TIAs and ADCs. The current data is processed in MCU and then transferred to the computer, where V_m_ is calculated and compared with V_th_. Once V_m_ exceeds V_th_, a fire event occurs and V_m_ will be cleared to zero. If the fire spike coincides with the reference random signal, the neuron will enter the depression stage. The computer sends the instructions to control DACs for selecting the proper signals, acting as the MUX. DAC of pre-neuron sends V_g_ according to the noise signal to WL, and that of post-neuron generates V_reset_ to SL and makes BL grounded. When the RESET spike overlaps with the noise spike, the device will be RESET to LGS. Otherwise, if the fire spike is not superimposed with a reference random signal, the neuron will turn to the potentiation stage. V_g_ is generated according to the input signal. V_set_ is sent to BL and SL is switched to the ground. When the SET spike overlaps with the input spike, the device will be SET to HGS. In other cases, the neuron remains in the accumulation stage. After training, the conductance at the final epoch is recorded as the learned weight. Note that the weight update is performed without write-verify, so there exists device variation as shown in Figure 3b. Figure 3c presents the measured and simulated results of SRDP characteristics. The frequency of the input signal is normalized by 1 MHz. For each frequency point, the weight is the mean of 300 times’ experiments after 100 training epochs. The outcome of measurement agrees with that of simulation. Because P_g_ is 0.5, the initialized weight is about 40.0μS. When the input frequency is higher than 0.3, the synapse experiences an enhancement process, otherwise, synaptic depression is triggered. The result shows that the relationship between the trained weights and the frequency of input signals is identical to the biological SRDP phenomenon, where LTP (LTD) is achieved with a high (low) frequency of input signal [37,38].

## 4. Online Unsupervised Learning of SRDP Network

We partition a 196 × 10 area of the array to construct a single-layer, fully-connected network consisting of 196 pre-neurons, 1960 synapses and 10 post-neurons. Ten handwritten digits from the MNIST data set [53] are selected. The input images are rescaled to 14 × 14 pixels to match the size of the array and then binarized. The input values of each image are unrolled into 196 × 1 vectors and then mapped to signals with different frequencies. Before training, the devices are initialized into HGS with the probability P_g_. The training parameters and the corresponding definitions are listed in Table 1, optimized by the fire-properly principle in ref. [4]. When the training starts, the accumulation process would be conducted first. DACs corresponding to the pattern pixels in the digit region send input signals with the same frequency P_in_ but different temporal sequences to WLs, and those within the background region generate signals with low-frequency P_b_. For the input signals at a high level, the corresponding transistors in the same row will be switched on. Meanwhile, V_s_ is applied to BLs of all post-neurons. The currents sharing the same column are integrated according to Kirchhoff’s current law. Due to the random distribution of initialized weights, post-neurons sharing the same input signals will have different accumulation speeds of membrane voltage and compete with each other. PC compares every membrane voltage with V_th_. Once any V_m_ exceeds V_th_, the corresponding post-neuron will experience weight update. No matter which post-neuron becomes the winner, V_m_ of all the post-neurons will be cleared to zeros. If the reference random signal with the frequency P_r_ is at a high level, the system will be in the depression stage. Different noise signals with certain rates will be generated by DACs. The post-neuron whose V_m_ exceeds V_th_ will send a RESET spike to SL, indicating that only the winner experiences the depression. The weight of the synapses connected to the winner will be tuned according to the noise signal. If the reference random signal is at a low level, the system will be in the potentiation stage and the synapses of the winner will have a certain probability to be enhanced. During the training process, the images are forwarded to the pre-neurons in sequence and each image holds for 600 training epochs.

**Figure 4:**
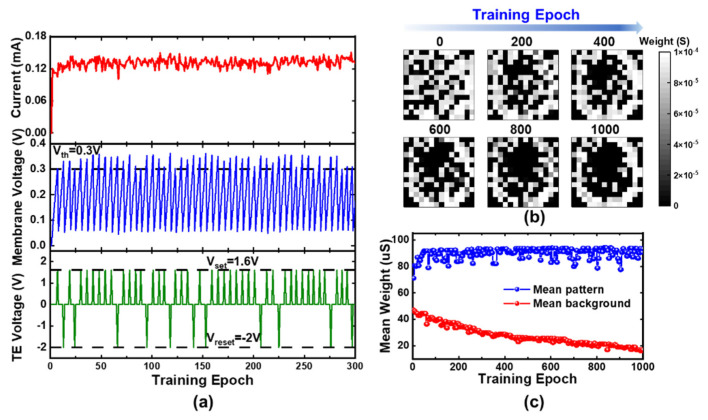
Hardware demonstration of the learning process for digit “0”. (**a**) Evolution of integral current (top), membrane voltage (middle) and the top electrode voltage (bottom); (**b**) Change process of the synapse weights; (**c**) Evolution of the mean weights in pattern (blue) and background (red) pixels.

Figure 4 shows the experimental learning process of digit “0”. In order to present more details, the training speed is slowed down by decreasing the parameter P_in_ and increasing the training epochs t_n_. In Figure 4a, the evolution of integral current, membrane voltage and the voltage of TE is shown during the first 300 epochs. The current across the synapses charges the capacitor of LIF post-neuron, contributing to the increase of membrane voltage. When the V_th_ is reached, a positive spike is transferred to TE and V_m_ will be cleared to zeros. Figure 4b shows the change of weights during the whole 1000 epochs, indicating that the weights in pattern regions get close to HGS and those in background regions tend to LGS. Figure 4c displays the evolution of mean weight corresponding to pixels in different regions. The results suggest that the potentiation (depression) occurs at high (low) frequency due to the larger probability for the weight to be enhanced (depressed), which is identical with the SRDP phenomenon [40,41,42]. 

**Figure 5:**
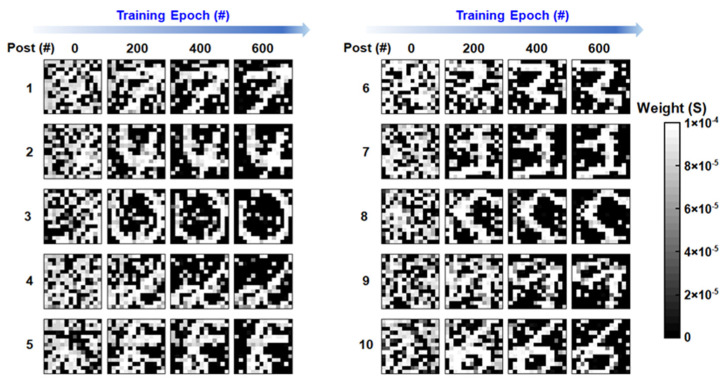
Experimental results of the learned synaptic weights for all digits.

**Figure 6:**
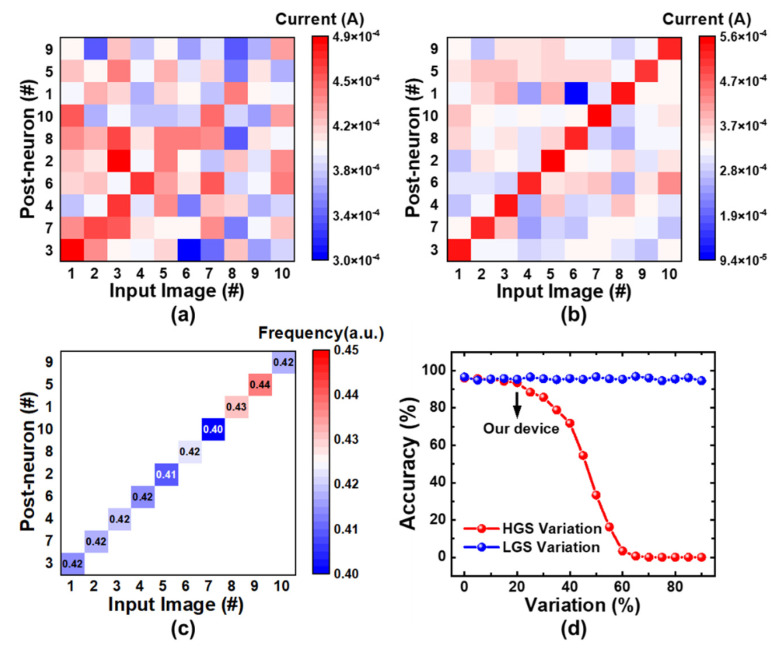
Experimental results of inference (**a**) before training and (**b**) after training. (**c**) Fire frequency during the training process. (**d**) Simulation results about the influence of the device variation on classification accuracy.

The learned synaptic weights of 10 handwritten digits are displayed in Figure 5. As the training goes on, the images are learned more clearly and the distinctions between inputs are enlarged, showing the learning ability of the SRDP network. The inference results before and after training are shown in Figure 6a,b. The post-neurons have been reordered, according to the fire sequence. The results show that the network fails to distinguish the inputs before training but succeeds to classify the digits after unsupervised learning. Figure 6c shows the normalized fire frequency for each digit. As can be seen in the result, one post-neuron fires for one digit, and 10 digits are learned by different post-neurons, indicating a successful classification. The gradual evolution process of the post-neuron dynamics in Figure 4 and the learned synaptic weights in Figure 5 is consistent with previous simulation outcomes in ref. [4], proving the SRDP network feasible. Considering that the synaptic weights are modulated in an unsupervised way without write-verify operations, the influence of the device variation on accuracy should be taken into account. Here, the accuracy is defined as the ratio of the number of successful classifications to the number of total measurements. We perform measurements 10 times and 9 of them succeed, which is in accordance with the simulation results as shown in Figure 6d. The result suggests that variation of HGS has more negative effect than that of LGS does. When HGS variation reaches 20%, the accuracy is 93.5%, which shows the strong robustness of the SRDP network.

**Figure 7:**
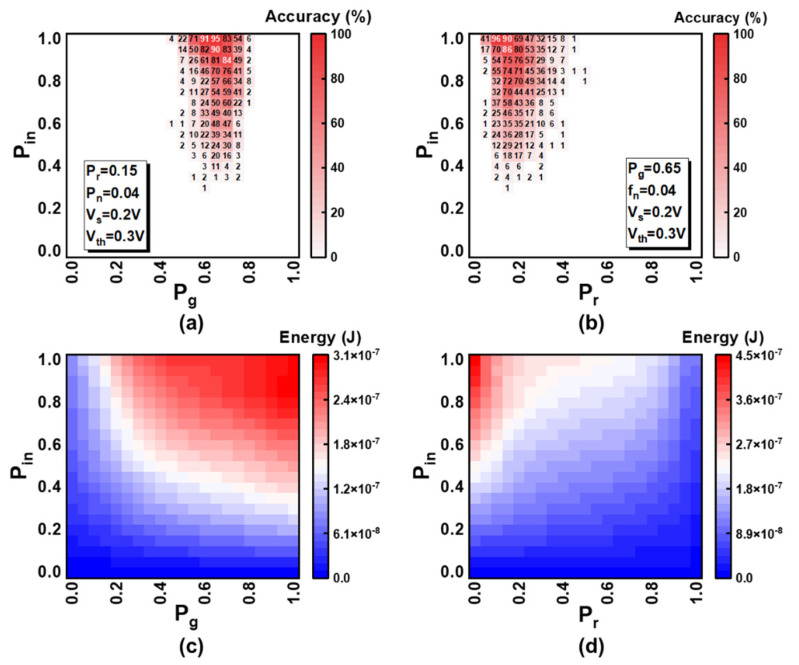
Simulation results of the network parameters’ impact. The influence of P_in_ and P_g_ on (**a**) the accuracy and (**c**) energy consumption. The influence of P_in_ and P_r_ on (**b**) the accuracy and (**d**) energy consumption.

The influence of the network parameters on training accuracy and energy consumption is simulated as shown in Figure 7. The accuracy is the most crucial standard for the network. P_in_ and P_g_ control the learning speed and have a great impact on accuracy, as shown in Figure 7a. These parameters cannot be too small, because the post-neurons will not learn images if they seldom fire. P_g_ cannot be too large, because when the number of set events for devices in HGS is smaller than that of reset events for devices in LGS, the training will fail, namely, forgetting is faster than learning for the neuron which should have been the winner at next epoch. The larger the P_in_ is, the higher the accuracy becomes in terms of this task. This is because the overlap rate between images is relatively small, thus, the images will be learned distinctly and the difference between images will be enlarged, if the training speed is fast. As for more complicated applications, the impact of P_in_ will be different, and all of the parameters should be re-optimized. P_r_ and P_n_ are also two critical parameters, and we only discuss the influence of P_r_ in Figure 7b. The product of P_r_ and P_n_ determines the probability of depression. If P_n_ is large, too many numbers of depression in pattern pixels may happen at a single epoch, which will make the learning fail in the worst case. However, if P_n_ is small, few reset events at one epoch will make the training process smoother and the probability of forgetting can remain unchanged by tuning the parameter P_r_. Thus, we adjust the probability of forgetting through the parameter P_r_ with the fixed slight value of P_n_. P_r_ cannot be too large in order to avoid catastrophic forgetting and cannot be too small because it will cause the winner to have no time resetting the devices in background pixels and continue to be the winner in the following learning period. The energy consumption shown in Figure 7c,d is calculated by integrating the current across the memristor array. With the increase of P_in_ and P_g_, the fire frequency is enlarged leading to more energy cost. Meanwhile, a larger probability of forgetting will decrease the fire frequency causing the reduction of power consumption. The outcomes indicate that the network parameters have a crucial impact on accuracy and energy consumption, and need to be fine-tuned and optimized for hardware demonstration.

## 5. Conclusions

In conclusion, we have constructed a neuromorphic hardware system with memristor synapses and CMOS neurons. The SRDP characteristic of memristor synapse is proved experimentally. The online unsupervised learning of 10 handwritten digits at a network level is successfully demonstrated by the SRDP algorithm with above 90% accuracy. The proposition of a bio-inspired SRDP algorithm and the construction of a neuromorphic hardware system paves the way towards the realization of large-scale and highly efficient neuromorphic systems.

## Figures and Tables

**Table 1:** The optimized network parameters.

Parameter	Definition	Value	Unit
V_s_	Constant voltage to the top electrode	0.2	V
V_th_	Threshold of the membrane voltage	0.3	V
P_g_	Probability to be in HGS of synaptic weights in the initial state	0.65	a.u.
P_r_	Frequency of the reference random signal	0.15	a.u.
P_n_	Frequency of the noise signal	0.04	a.u.
P_in_	Frequency of the input signal in the pattern pixels	1	a.u.
P_b_	Frequency of the input signal in the background pixels	0	a.u.
t_n_	Training epoch of each image	600	#

## Data Availability

Not applicable.
